# The pararectus approach—a versatile option in pelvic musculoskeletal tumor surgery

**DOI:** 10.1186/s13018-019-1275-x

**Published:** 2019-07-23

**Authors:** Christophe Kurze, Marius Johann Baptist Keel, Attila Kollár, Klaus Arno Siebenrock, Frank Michael Klenke

**Affiliations:** 1 Department of Orthopedic Surgery, Inselspital, Bern University Hospital, CH-3010 Bern, Switzerland; 2 Department of Medical Oncology, Inselspital, Bern University Hospital, CH-3010 Bern, Switzerland

**Keywords:** Complications, Pararectus approach, Pelvic tumor, Resection, Sarcoma

## Abstract

**Background:**

Pelvic tumors are usually resected through the utilitarian pelvic incision, an extended ilioinguinal/iliofemoral approach. The pararectus approach, an intrapelvic anatomical approach with extraperitoneal access to the pelvis, has been established previously for the treatment of pelvic and acetabular fractures. However, it has not been used to address pelvic tumors. The study aimed at investigating the feasibility of this approach for pelvic tumor surgery and the possibilities of combining this approach with standard approaches to the hip joint.

**Methods:**

Thirteen patients that underwent pelvic tumor resections were retrospectively reviewed. Tumor resections were performed through the pararectus (*n* = 10) or extended pararectus approach (*n* = 3). In six of those cases, the pararectus approach was combined with extrapelvic approaches including the modified Gibson (*n* = 4), the Kocher-Langenbeck (*n* = 1), and the trochanteric flip approach (*n* = 1). The mean follow-up was 32.6 ± 9.1 months.

**Results:**

In all cases, the tumor resections were carried out according to the preoperative plan. In seven of 13 cases, wide resections were performed; six of 13 cases were planned close resections. Four cases of major complications were observed (vascular injury, deep infection, iliac vein thrombosis, total hip arthroplasty dislocation). Minor complications were observed in two cases. One tumor recurred locally. At the final follow-up, 10 patients were alive, eight of those without evidence of disease.

**Conclusion:**

The study demonstrated the suitability of the pararectus approach for pelvic tumor resections. The possibility to combine the approach with standard approaches to the hip joint allowed for single-stage reconstructions of the pelvis and the hip joint without sacrificing surgical margins and function. The pararectus approach is a versatile option adding to the established approaches for musculoskeletal tumor surgery of the pelvis.

## Background

Surgical management of pelvic bone and soft tissue tumors is one of the most complex fields in musculoskeletal oncology and is associated with a high risk of complications. The utilitarian pelvic incision and its modifications have been well-established for pelvic tumor surgery [1–8]. However, as an extrapelvic approach, it provides limited access to the iliac fossa and the intrapelvic neurovascular bundles. The abdominoinguinal incision, which has been described by Karakousis, achieves good exposure and control of the vital intrapelvic structures but involves the disadvantages of groin dissection and opening of the peritoneal cavity [9].

The pararectus approach, an intrapelvic anatomical approach with extraperitoneal access to the pelvis, has been established previously for the treatment of pelvic and acetabular fractures [10–12]. The approach avoids the medial flank of the utilitarian approach and can be extended distally over the inguinal fold. Standard approaches to the hip joint including the Kocher-Langenbeck and the modified Gibson approach can be combined with the incision without creating a skin flap prone to wound healing complications. Here we report the first 13 cases of musculoskeletal tumors of the pelvis resected through the pararectus approach. The study aimed at investigating the feasibility of this approach for pelvic tumor surgery and at illustrating possibilities of combining this approach with standard approaches to the hip joint.

## Patients and methods

Between 2010 and 2015, 16 patients were treated for bone and soft tissue tumors of the pelvis at our institution. Out of those, the first 13 consecutive patients (6 males/7 females, Table 1) that underwent musculoskeletal tumor surgery of the pelvis via the pararectus approach were retrospectively reviewed. The mean age was 53.4 ± 19.8 years (range 21–76 years). Eight patients were treated for a malignant tumor (chondrosarcoma *n* = 2, high-grade undifferentiated sarcoma *n* = 2, high-grade osteogenic sarcoma *n* = 1, radiation-induced osteosarcoma *n* = 1, myxofibrosarcoma *n* = 1, malignant solitary fibrous tumor (SFT) *n* = 1); tumors were graded G1 (*n* = 2), G2 (*n* = 2), and G3 (*n* = 4) according to the FNCLCC classification. Five patients were treated for a benign (lipoma, *n* = 3) or a locally aggressive tumor (desmoplastic fibroma/desmoid tumor, *n* = 2). Seven tumors were primary bone tumors and six were soft tissue tumors.

**Table 1:** Patient and disease characteristics

Patient no.	Age at surgery	Sex	Tumor	Location	Approach	Resection	Blood loss (ml)	Surgery time (min)	Major complications	Minor complications	Follow-up (months)	Disease status
1	22	M	Chondrosarcoma	Ilium	PR	R0	n.s.	150			30	NED
2	65	M	Myxofibrosarcoma	Iliacus muscle	PR	R0	700	165		Scar hernia	48	NED
3	49	F	Lipoma	Intrapelvic, buttock	PR, Gibson	R1	310	270			49	NED
4	50	M	High-grade undifferentiated sarcoma	Pubis, acetabulum	Extended PR	R0	5000	840	THA dislocation, Infection		14	DOD
5	71	F	Lipoma	Intrapelvic, Buttock	PR, Gibson	R0	600	190			32	NED
6	61	F	Lipoma	Intrapelvic, thigh	Extended PR	R1	300	240		Meralgia paraesthetica	36	NED
7	53	M	Recurrent chondrosarcoma	Ischium, retroperitoneum	PR, Gibson	R1	3300	300			29	AWD
8	76	M	High-grade undifferentiated sarcoma	Retroperitoneum	PR	R0	3500	300			8	DOD
9	67	M	Malignant solitary fibrous tumor	Intrapelvic, buttock	PR, Kocher-Langenbeck	R1	18,000	720	Vessel lesion, mass transfusion, iliac vein thrombosis		27	AWD
10	25	F	Desmoplastic fibroma of bone	Pubis, acetabulum	PR, trochanteric osteotomy	R0	2500	600			25	NED
11	65	F	Radiation induced osteosarcoma	Pubis, ilium	Extended PR	R0	1700	420	Infection		4	DOD
12	54	F	High-grade osteogenic sarcoma	Pubis, ischium	PR, Gibson	R0	2000	660	Infection		26	NED
13	21	F	Desmoplastic fibroma of bone	Pubis	PR	R1	800	210			24	NED

*PR* pararectus approach, *n.s* not specified, *NED* no evidence of disease, *AWD* alive with disease, *DOD* dead of disease

Preoperative workup included medical history, clinical examination, and routine blood tests. Plain radiographs and MRI scans of the pelvis were obtained in each case. Patients with primary bone tumors received additional CT scans of the pelvis. In patients with a malignant tumor on biopsy, a thoracic and abdominal staging CT was performed; this confirmed localized tumor disease in all patients. Biopsy was done in every patient either by image-guided core needle biopsy or open biopsy. One patient received neoadjuvant radiotherapy. Adjuvant therapy was performed in two patients; irradiation and chemotherapy in one case each.

**Fig. 1:**
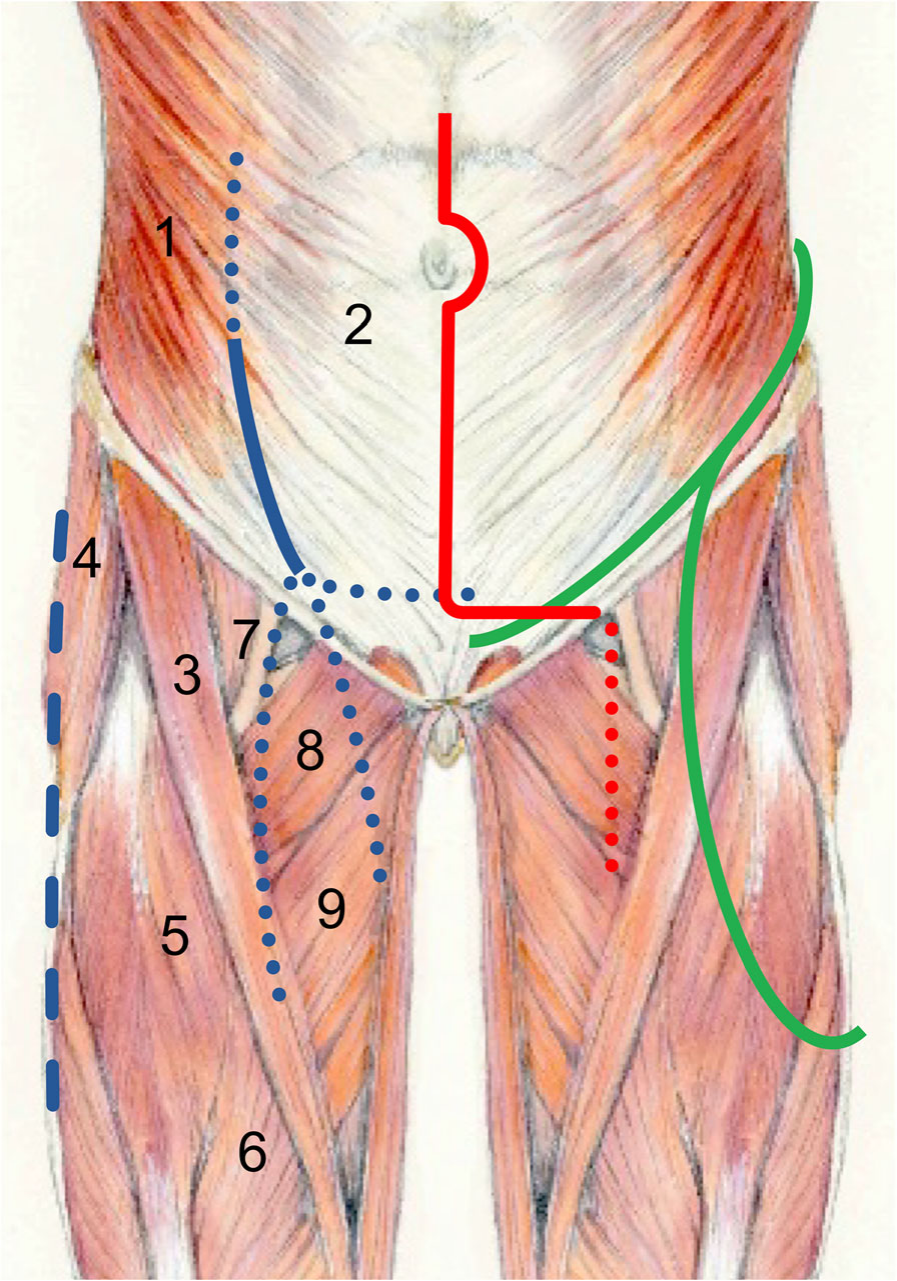
Schematic drawing of the muscular anatomy of the anterior abdominal wall and thigh. Superimposed skin incisions of the pararectus approach (blue), the utilitarian incision (green), and the abdominoinguinal incision (red). The primary incisions are shown by solid lines, extensions by dotted lines. The pararectus approach may be combined with lateral approaches to the hip and thigh (indicated with blue dashed line). 1. Obliquus externus muscle. 2. Aponeurosis of obliquus externus muscle with underlying rectus abdominis muscle. 3. Sartorius muscle. 4. Tensor fascia lata muscle. 5. Rectus femoris muscle. 6. Vastus medialis muscle. 7. Iliopsoas muscle and tendon. 8. Pectineus muscle. 9. Adductor longus muscle

The pararectus approach was performed as described in detail by Keel et al. for the treatment of acetabular fractures [11]. In brief, patients were placed in supine position with the hip flexed slightly. An incision was directed along the lateral border of the rectus abdominis (Fig. 1). The rectus sheath was incised at the lateral boarder after deep dissection and incision of the anterior abdominal wall. The transversalis fascia was visualized and entered in the extraperitoneal space without harming the peritoneum, the bladder, and the epigastric and external iliac vessels.

**Fig. 2:**
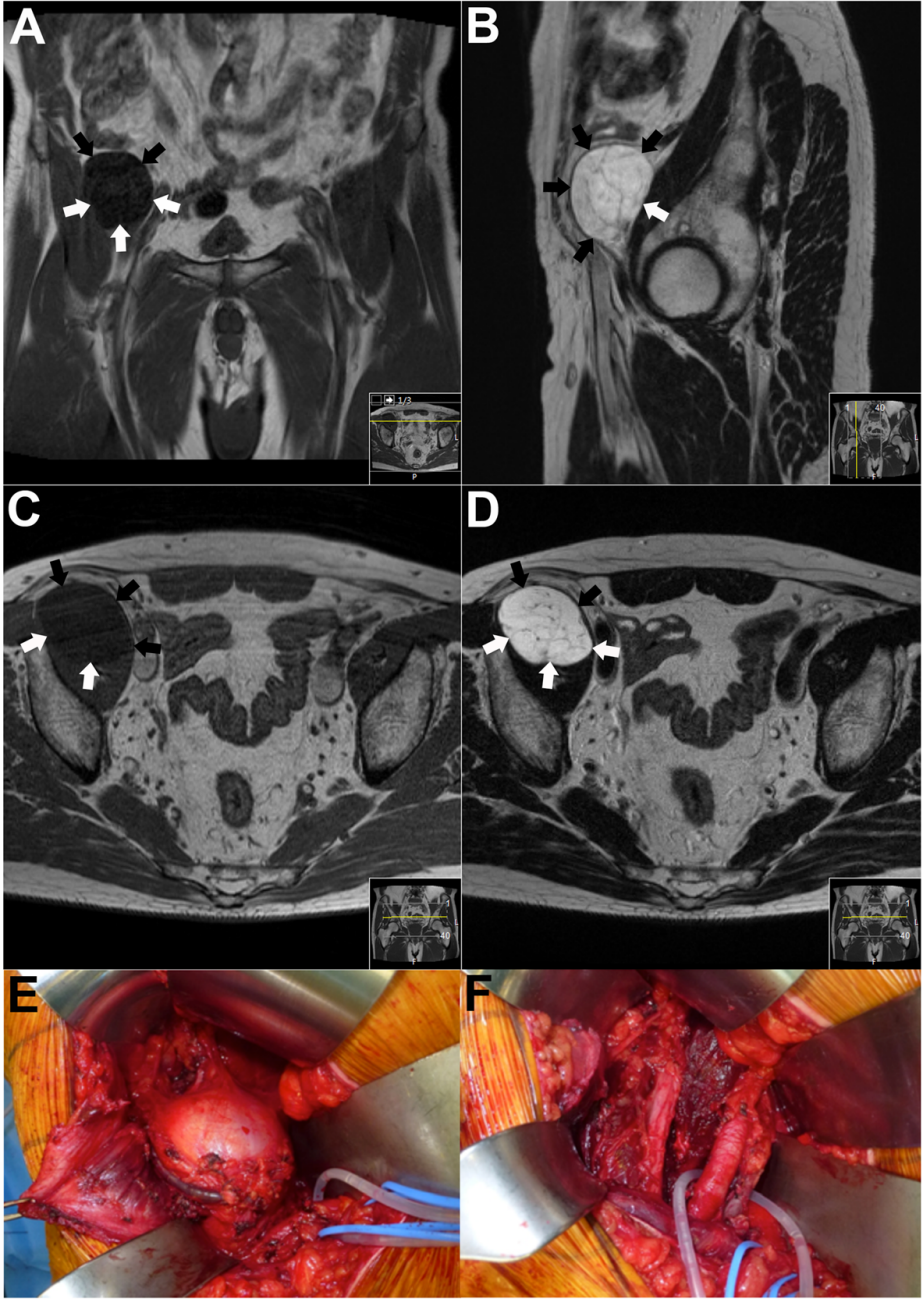
MRI imaging and intraoperative photo documentation of a 65-year-old male patient who presented with a soft tissue tumor situated in the right iliac muscle measuring approximately 5.7 × 4.8 × 4.6 cm ((**a**–**d**), tumor margins marked with arrows). The tumor was hypointens on T1 (**a**, **c**) and hyperintens on T2 (**b**, **d**) images. Core needle biopsy confirmed a low-grade (G1) myxofibrosarcoma. As the tumor was close to the external iliac vessels a marginal resection was performed. The photographs demonstrate the intraoperative situation before (**e**) and after (**f**) tumor resection; the external iliac vessels are tagged (**f**)

Tumor resections were performed through the pararectus approach (*n* = 10, Fig. 2) or through an extended pararectus approach (*n* = 3) with distal extension through the inguinal canal. In six cases, the pararectus approach was combined with extrapelvic approaches to the hip including the Kocher-Langenbeck approach (*n* = 1), trochanteric flip approach (*n* = 1), and modified Gibson approach (*n* = 4). Seven tumor resections were planned as wide resections and six as close or intralesional resections. Planned close resections were carried out in lipomas (*n* = 3). Planned intralesional resections were performed in desmoplastic fibroma of the bone (*n* = 1), recurrent low-grade chondrosarcoma (*n* = 1), and a malignant SFT (*n* = 1). In the desmoplastic fibroma of the bone, wide resection versus intralesional resection was discussed with the patient. As the tumor was adjacent to the antero-inferior acetabulum, resection with negative margins would have resulted in partial resection of the hip joint. To minimize functional impairment, decision for intralesional resection with extended curettage and heat ablation was made based on recent reports. The malignant SFT showed tumor growth through the greater sciatic notch towards the posterior thigh with close proximity of the tumor to vital neurovascular structures. Wide resection would have meant to sacrifice the sciatic nerve and was refused by the patient. In the recurrent low-grade chondrosarcoma, the patient preferred limited surgery with preservation of function to a wide tumor resection involving amputation of the penis.

The realization of the planned resection served as the primary outcome parameter. Secondary outcome parameters were major and minor complications and duration of the intervention. Major complications were defined as complications requiring a second surgery. The minimum follow-up was 24 months. The mean follow-up was 32.6 ± 9.1 months (24–49 months).

## Results

In all cases, the tumor resections were carried out according to the preoperative plan. In all wide resections, R0 resection status was confirmed histologically. Planned close resections of lipomas resulted in R0 resection status in one and R1 resection status in two cases, respectively. Intralesional resections demonstrated positive histological margins in all three cases.

Blood loss was 3225 ± 4881 ml (300–18,000 ml). Mean duration of the surgeries was 6.5 ± 3.9 h (2.5–14 h). Four major complications were observed in four cases. Those were mass transfusion due to injury of tumor invading blood vessels, deep infection (*n* = 3), iliac vein thrombosis (*n* = 1), and total hip arthroplasty dislocation (*n* = 1). All major complications were controlled with the same or repeat surgical intervention. Two cases of minor complications were observed (scar hernia (*n* = 1), meralgia paraesthetica (*n* = 1)).

In four cases with primary bone tumors, an internal hemipelvectomy was performed [PI, II, III, H1 (*n* = 3), PII, III, H1 (*n* = 1), according to the classification of Enneking [13]]. In three of these four patients, a reconstruction of the osseous pelvis was performed. One patient was reconstructed with a bone allograft. Two patients received a custom 3D-printed titanium implant (Materialise Inc., Leuven, Belgium). In all three cases, the pelvic reconstruction was combined with a total hip arthroplasty. In the patient with a radiation induced high-grade osteosarcoma, no reconstruction of the pelvis was performed due to early post-operative infection and subsequent rapid onset of systemic disease. The patient died 4 months after the index surgery.

At the final follow-up, 10 patients were alive. Eight patients did not have any evidence of disease and two patients were alive with disease. Of the latter, the patient treated for recurrent chondrosarcoma developed another local recurrence after 13 months and the patient with malignant SFT was diagnosed with systemic disease, i.e., lung metastases 27 months after the index procedure. Another three patients deceased due to systemic disease 4, 8, and 14 months after the primary tumor resection, respectively.

## Discussion

The present consecutive case series aimed at investigating the feasibility of the pararectus approach for pelvic tumor surgery. The investigation of the first 13 cases of tumor patients treated with this surgical approach has clear limitations due to the low number of cases included in the study and the heterogeneity of the study group. On the other hand, the heterogeneity of the study group indicates that the approach may be applicable to a wide variety of bone and soft tissue tumors of the pelvis.

Due to the complex three-dimensional anatomy and the proximity to vital neurovascular structures, a good visualization of the operative field is essential in pelvic tumor surgery [1]. The present study showed that the pararectus approach achieved good exposure of the entire hemipelvis including the pubis, the acetabulum, the sacro-iliac joint, the spermatic cord, and the neurovascular structures (Table 2). All tumor resections including internal hemipelvectomies could be carried out according to the preoperative plan. Distal extension of the approach and its combination with approaches to the hip joint enabled us to address complex tumors with extrapelvic extension. It seems most likely that hemipelvectomies involving the sacrum (type P IV resection) are feasible due to the possibility to extend the pararectus approach proximally [15]. However, there was no such case included in this case series. On the other hand, the pararectus approach may not be suitable in obese patients and patients presenting with bowel obstruction and abdominal distension [11].

**Table 2:** Advantages and limitations of the pararectus approach compared to established approaches in pelvic tumor surgery

	Indications	Advantages	Limitations
Pararectus approach	Pelvic bone and soft tissue tumors	• Avoids extensive soft tissue flaps prone to wound healing problems • Uncomplicated medial, proximal and distal extension • Facilitates addressing periacetabular bone tumors, especially when intrapelvic soft tissue expansion is present • Good control of intra- and extrapelvic neurovascular structures	• Limited exposure in obese patients and cases with bowel obstruction • Mobilization of large tumors restricted due to avoidance of soft tissue flaps
Utilitarian incision and modifications [3, 4, 6–8]	Pelvic bone tumors	• Wide exposure of the entire osseous pelvis through creation of soft tissue flaps •Wide exposure facilitates mobilization of large tumors and reconstruction of osseous pelvis	• Increased risk of wound healing disorders due to T-shaped skin incision and soft tissue flaps • Limited exposure of intrapelvic neurovascular structures, especially in cases with large soft tissue masses (exposure through ilioinguinal window)
Abdominoinguinal incision [5, 9, 14]	Pelvic bone and soft tissue tumors	• Wide exposure of intrapelvic organs through creation of soft tissue flaps • Uncomplicated proximal and distal extension • Facilitates addressing periacetabular bone tumors, especially when intrapelvic soft tissue expansion is present • Good control of intra- and extrapelvic neurovascular structures	• Increased risk of wound healing disorders due to (double) L-shaped skin incision and soft tissue flaps • Entering peritoneal cavity disturbs a natural barrier against tumor dissemination and is associated with an increased risk of intraabdominal adhesions

In this study, the pararectus approach was applied to resections of bone and soft tissue tumors of the pelvis. The standard exposure for tumor resections of the osseous pelvis involves the utilitarian incision first described by Enneking and Dunham [8]. Through this extended ilioinguinal approach with reflection of a large soft tissue flap, the buttock, the sciatic notches, the ischium, and the proximal femur can be exposed. The symphysis is exposed through extension of the incision along the medial half of the inguinal ligament [8]. Several modifications of the utilitarian approach attempting to improve surgical exposure and minimize the risk of neurovascular complications have been described [3–7]. Lackman et al. [7] described a T-shaped incision where the “T” of the incision is located more laterally than the turning point of the original single incision. The T-incision provides a more extensile anterior to posterior exposure. Furthermore, Karakousis described the abdominoinguinal incision previously for resections of pelvic soft tissue and bone tumors [5, 9, 14]. Similar to the pararectus approach, the abdominoinguinal approach achieves good control over the intrapelvic neurovascular structures and the spermatic cord. However, the approach uses a midline incision with transverse lateral extension to and over the inguinal fold resulting in a skin flap that may be prone to wound healing problems.

Complication rates of pelvic tumor surgeries are high. The complication rate of tumor resections performed through the utilitarian approach has been reported to range from 31 to 60% [7, 16–18]. Infections and wound healing problems account for the majority of complications and have been shown to occur in 7–50% and 13–29%, respectively [7, 8, 17–21]. It has been hypothesized that the factors contributing to these complications are the large size of the surgical wound and limited perfusion of the fasciocutaneous flaps [7]. In our series, we did not observe wound healing complications. This may be because the pararectus approach is combined with approaches on the lateral side of the femur to the hip when extensive exposures of the pelvis, the hip, and the thigh are required. The combination of two approaches avoids a long, curved single incision or a T-shaped incision and the development of a fasciocutaneous flap. Despite the decreased wound healing complications, the rate of deep infections was not reduced. Deep infections were observed in four out of 13 patients, which is in accordance with the existing literature. We believe that factors other than wound healing have a stronger effect on the risk of deep infections such as surgery time, radio-/chemotherapy, and extent of the tumor surgery. Indeed, three out of four infections occurred in patients receiving neoadjuvant chemotherapy and/or undergoing hemipelvectomy. This is in accordance with Angelini et al. [22] who showed that the only positive predictor in developing an infection was the pelvic reconstruction in patients undergoing pelvic bone tumor resections. In the present study, blood loss was higher than previously reported in pelvic tumor surgery. However, the blood loss and surgery time in our case series were mainly influenced by one case, in which intraoperative vascular damage resulted in massive blood loss.

## Conclusions

The pararectus approach is a versatile option adding to the established approach for musculoskeletal tumor surgery of the pelvis. Studies with longer follow-up and larger case numbers are needed to further validate these encouraging results.

## Data Availability

The datasets used and/or analyzed during the current study are available from the corresponding author on reasonable request.

## References

[1] McGoldrick NP, Butler JS, Lavelle M, Sheehan S, Dudeney S, O’Toole GC (2016). Resection and reconstruction of pelvic and extremity soft tissue sarcomas with major vascular involvement: current concepts. World J Orthop.

[2] Mavrogenis AF, Soultanis K, Patapis P, Guerra G, Fabbri N, Ruggieri P, Papagelopoulos PJ (2012). Pelvic resections. Orthopedics.

[3] Steel HH (1978). Partial or complete resection of the hemipelvis. An alternative to hindquarter amputation for periacetabular chondrosarcoma of the pelvis. J Bone Joint Surg Am.

[4] Mankin HJ, Hornicek FJ (2005). Internal hemipelvectomy for the management of pelvic sarcomas. Surg Oncol Clin N Am.

[5] Karakousis CP, Emrich LJ, Driscoll DL (1989). Variants of hemipelvectomy and their complications. Am J Surg.

[6] Bickels J, Malawer M. Pelvic resections (internal hemipelvectomies). In: Malawer MM, Sugarbaker PH, editors. Musculoskeletal cancer surgery: Treatment of sarcomas and allied diseases. Dordrecht: Springer; 2001. p. 405–14.

[7] Lackman RD, Crawford EA, Hosalkar HS, King JJ, Ogilvie CM (2009). Internal hemipelvectomy for pelvic sarcomas using a T-incision surgical approach. Clin Orthop Relat Res.

[8] Enneking WF, Dunham WK (1978). Resection and reconstruction for primary neoplasms involving the innominate bone. J Bone Joint Surg Am.

[9] Karakousis CP (2000). Abdominoinguinal incision and other incisions in the resection of pelvic tumors. Surg Oncol.

[10] Keel MJ, Tomagra S, Bonel HM, Siebenrock KA, Bastian JD (2014). Clinical results of acetabular fracture management with the Pararectus approach. Injury.

[11] Keel MJ, Ecker TM, Cullmann JL, Bergmann M, Bonel HM, Buchler L, Siebenrock KA, Bastian JD (2012). The Pararectus approach for anterior intrapelvic management of acetabular fractures: an anatomical study and clinical evaluation. J Bone Joint Surg Br.

[12] Bastian JD, Savic M, Cullmann JL, Zech WD, Djonov V, Keel MJ (2016). Surgical exposures and options for instrumentation in acetabular fracture fixation: pararectus approach versus the modified Stoppa. Injury.

[13] Enneking W, Dunham W, Gebhardt M, Malawar M, Pritchard D (1990). A system for the classification of skeletal resections. Chir Organi Mov.

[14] Karakousis CP (1998). The abdominoinguinal incision: the equivalent of thoracoabdominal incision for the lower quadrants of the abdomen. J Surg Oncol.

[15] Fraser RD, Gogan WJ (1992). A modified muscle-splitting approach to the lumbosacral spine. Spine (Phila Pa 1976).

[16] Asavamongkolkul A, Pimolsanti R, Waikakul S, Kiatsevee P (2005). Periacetabular limb salvage for malignant bone tumours. J Orthop Surg (Hong Kong).

[17] Donati D, Giacomini S, Gozzi E, Ferrari S, Sangiorgi L, Tienghi A, DeGroot H, Bertoni F, Bacchini P, Bacci G, Mercuri M (2004). Osteosarcoma of the pelvis. Eur J Surg Oncol.

[18] Wirbel R. J., Schulte M., Mutschler W. E. (2001). Surgical Treatment of Pelvic Sarcomas. Clinical Orthopaedics and Related Research.

[19] Apffelstaedt JP, Driscoll DL, Karakousis CP (1995). Partial and complete internal hemipelvectomy: complications and long-term follow-up. J Am Coll Surg.

[20] Baliski CR, Schachar NS, McKinnon JG, Stuart GC, Temple WJ (2004). Hemipelvectomy: a changing perspective for a rare procedure. Can J Surg.

[21] Kollender Y, Shabat S, Bickels J, Flusser G, Isakov J, Neuman Y, Cohen I, Weyl-Ben-Arush M, Ramo N, Meller I (2000). Internal hemipelvectomy for bone sarcomas in children and young adults: surgical considerations. Eur J Surg Oncol.

[22] Angelini A, Drago G, Trovarelli G, Calabro T, Ruggieri P (2014). Infection after surgical resection for pelvic bone tumors: an analysis of 270 patients from one institution. Clin Orthop Relat Res.

